# SLEEVE GASTRECTOMY AND FUNDOPLICATION AS A SINGLE PROCEDURE IN PATIENTS WITH OBESITY AND GASTROESOPHAGEAL REFLUX

**DOI:** 10.1590/0102-6720201700030012

**Published:** 2017

**Authors:** Juan Pablo LASNIBAT, Italo BRAGHETTO, Luis GUTIERREZ, Felipe SANCHEZ

**Affiliations:** 1 Department of Surgery, Faculty of Medicine, University Hospital Dr José J. Aguirre, Santiago, Chile.

**Keywords:** Obesity, Bariatric Surgery, Gastroesophageal Reflux, Fundoplication, Sleeve gastrectomy

## Abstract

**Background:**

Bariatric surgery in Chile has seen an exponential increase in recent years, especially in sleeve gastrectomy. Its use is currently discussed in patients suffering from gastroesophageal reflux disease. Different options have been considered for the management of these patients but up to now laparoscopic Roux-en-Y gastric bypass seems to be the best option. Sleeve gastrectomy plus concomitant fundoplication or hiatal hernia repair also has been suggested in patients having reflux or small hiatal hernia.

**Aim:**

To present a cohort of obese patients with gatroesophageal reflux undergoing this procedure, which seeks to provide the benefits of both laparoscopic gastric sleeve (LSG) and antireflux surgery focused on the evaluation of presence of reflux and BMI after surgery, and to compare the result observed in this cohort with a previous group of obese patients without reflux submitted to sleeve gastrectomy alone.

**Methods:**

Retrospective case series in 15 patients who underwent this surgery between the years 2003 and 2012. Clinical records were analyzed and values ​​of 24 hr pH monitoring, esophageal manometry and clinical outcome were recorded. Results were compared to a previous series of patients who underwent LSG. No statistical analyses were made.

**Results:**

Group A consisted of 15 patients submitted to LSG plus fundoplication. 93% (n=14) were female. Mean age was 46.2 years. Mean preoperative body mass index (BMI) was 33.9. All patients had altered pH monitoring and manometry preoperatively. There was one minor complication corresponding to a seroma. There was no perioperative mortality. Group B consisted of 23 obese patients who underwent LSG. These patients developed de novo reflux, hypotensive LES and esophagitis after the surgery. Group A patients showed improvement in esophageal pH monitoring and manometry at three months. During long-term follow-up, six underwent revision surgery, four for weight regain, one regained weight associated with symptomatic reflux, and one underwent re-intervention for reflux.

**Conclusions:**

Good results are observed in the short-term follow up in both reflux resolution and weight loss. Nevertheless, results at long term are discouraging, with 53.3% of the patients requiring revision surgery during follow-up.

## RESUMO

**Racional:**

A cirurgia bariátrica no Chile tem visto aumento exponencial nos últimos anos, especialmente na gastrectomia vertical. Atualmente, o seu uso é discutido em pacientes que sofrem de refluxo gastroesofágico. Diferentes opções foram consideradas para o gerenciamento desses pacientes, mas até agora o bypass gástrico em Y-de-Roux laparoscópico parece ser a melhor opção. A gastrectomia vertical mais fundoplicatura concomitante ou o reparo da hérnia hiatal também foi sugerido em pacientes com refluxo ou hérnia hiatal pequena.

**Objetivo:**

Apresentar uma coorte de pacientes obesos com refluxo gatroesofágico submetidos a esse procedimento, que busca proporcionar os benefícios da gastrectomia vertical laparoscópica (LSG) e da operação antirrefluxo focada na avaliação da presença de refluxo e IMC após a operação, e comparar a o resultado observado nesta coorte com um grupo anterior de pacientes obesos sem refluxo submetido somente à gastrectomia vertical.

**Métodos:**

Série de casos retrospectivos em 15 pacientes submetidos à essa operação entre os anos de 2003 e 2012. Os registros clínicos foram analisados ​​e os valores de monitoramento de pH 24 h, manometria esofágica e desfecho clínico foram registrados. Os resultados foram comparados com uma série anterior de pacientes submetidos à LSG. Não foram feitas análises estatísticas.

**Resultados:**

O grupo A consistiu em 15 pacientes submetidos a LSG mais fundoplicatura e 93% (n=14) eram mulheres. A idade média foi de 46,2 anos. O índice de massa corporal pré-operatório médio (IMC) foi de 33,9. Todos os pacientes apresentaram monitoração de pH e manometria pré-operatória alteradas. Houve uma complicação menor correspondente a um seroma. Não houve mortalidade perioperatória. O grupo B consistiu de 23 pacientes obesos submetidos à LSG. Esses pacientes desenvolveram refluxo de novo, LES hipotensos e esofagite após a operação. Os pacientes do grupo A apresentaram melhora no monitoramento e manometria do pH esofágico aos três meses. Durante o seguimento em longo prazo, seis foram submetidos à operação revisional, quatro por recuperação de peso, um por peso recuperado associado a refluxo sintomático e um submetido à reintervenção por refluxo.

**Conclusões:**

Bons resultados são observados no seguimento em curto prazo na resolução de refluxo e perda de peso. No entanto, os resultados em longo prazo são desencorajadores, pois 53,3% dos pacientes necessitaram de operação revisional durante o acompanhamento.

## INTRODUCTION

Bariatric surgery in Chile and in the world has experienced a significant increase over the last decade. There has been a trend in favor of laparoscopic sleeve gastrectomy (LSG), which represents about 70% of the bariatric procedures. In recent times this surgery was most commonly performed in patients with body mass index (BMI) under 35^1^ ^,^ ^37^. These patients are an excellent group to perform this procedure^33^; however, there is no consensus about its effects when patients suffer from gastroesophageal reflux disease (GERD). There are conflicting studies on the relationship between sleeve gastrectomy (SG) and GERD in the literature. Multiple publications have reported changes in resting pressure of the lower esophageal sphincter (LES) after a SG, and multiple studies show an increase in GERD in the first year after surgery ^1,10,12,23,25,26,30,41^.

In obese patients who have concomitant GERD, our options are to perform laparoscopic Roux-en-Y gastric bypass as a primary operation (adding hiatal hernia repair if it is present). In our experience, after LSG, we have not observed improvement of reflux; on the contrary we have observed “de novo“ reflux and esophagitis in more than 20% of patients^4^. For this reason, LSG is not performed in patients with GERD in our center, and we prefer to opt for a laparoscopic Roux-en-Y gastric bypass. 

Obese patients with BMI of 35 kg/m^2^ or lower, are a special group, were overall LSG achieves excellent results in weight loss and resolution of comorbidities, but in patients with concomitant GERD (with or without hiatal hernia), this procedure alone doesn´t improve GERD. In this context we evaluated a group of obese patients with GERD in whom LSG plus Nissen fundoplication was employed, which seeks to provide the benefits of both SG and anti-reflux surgery. The results were compared with the results observed in a previous group of patients submitted to LSG alone.

The aim of this study was to assess postoperative outcomes of patients undergoing this surgery in order to evaluate late outcome, focused on BMI evolution and presence or not of GERD after surgery. This procedure should generate good weight loss measured in postoperative controls, in addition to improving the parameters in studies of esophageal pH monitoring and manometry.

## METHODS

The study was presented to ethical research committee of our hospital. All procedures performed in studies involving human participants were in accordance with the ethical standards of the institutional and/or national research committee and with the 1964 Helsinki declaration and its later amendments or comparable ethical standards. Informed consent was obtained from all individual participants included in the study. Patients gave their informed consent for surgery and follow-up. 

This study includes two groups of obese patients. A cohort of 15 patients (group A) undergoing LSG with Nissen fundoplication concomitantly performed due to preoperative GERD with reflux symptoms and erosive esophagitis confirmed by endoscopy, manometry and 24 hr pH monitoring. On the 15 patients studied, 93% (n=14) correspond to female. The mean age was 46.2 years. The mean preoperative BMI was 33.9±2.11 kg/m^2^. Patients had a very close follow up during the first year with clinical evaluation, postoperative endoscopy, manometry, and 24 h pH monitoring. The results were compared with another group of obese patients from a previous study, without reflux symptoms, erosive esophagitis or hiatal hernia (group B), composed by 23 patients, 12 women and 11 men with a mean age of 37.3 years (range 16-69), submitted to LSG without anti-reflux procedure or hernia repair because they did not present any expression of GERD. The BMI of this group of patients was 37.5±4.4 kg/m^2^ (30.3-56). Also clinical evaluation, manometry and 24 h pH monitoring was done in this group (Table 1).

**TABLE 1:** Demographics

	Group 1	Group 2
	n=15	n=23
Age (years)	46.2	37.3
Gender (female:male)	14:1	12:11
BMI (kg/m2)	33.9 ± 2.11	37.5 ± 4.4
Operating time (minutes)	157 ± 22.13	87 ± 15
Hospital stay (days)	4.6	2.6
Complications (%)	13.3% (2/15)	4.3% (1/23)
Deaths (n)	0	0

For the late follow-up, the majority of patients of both groups were controlled personally and others were contacted by telephone or email survey. 

**FIGURE 1:**
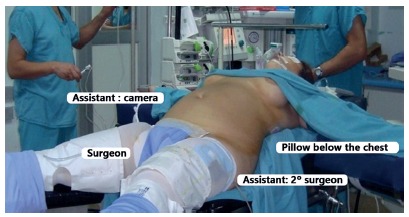
Patient´s position: Grassi´s position with the legs in abduction (French approach)

Regarding the surgical technique, the patient is positioned in Grassi´s position with French approach with the surgeon between the patient’s legs, with a first assistant to the left of the patient, and a second assistant to the right (Figure 1). Pneumoperitoneum is made in the upper abdomen area and a 12 mm trocar is positioned in the supraumbilical position. A 5 mm subxiphoid trocar is used for liver retraction. Two 10 mm additional trocars are positioned in left and right flank. An additional 5 mm trocar is positioned 10 mm lateral to the left flank.

**FIGURE 2:**
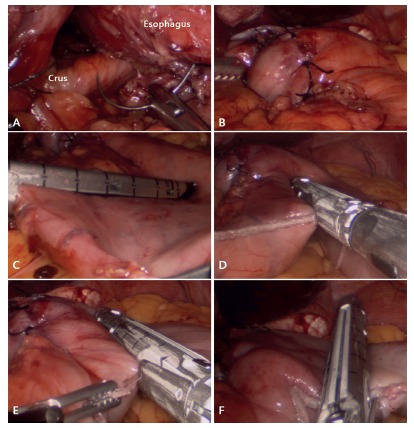
A) Clousure of diaphragmatic crus; B) Nissen fundoplication; C) sleeve gastrectomy starting 2-3cm from the pylorus; D) sleeve gastrectomy with body vertical transaction; E) transection of the upper part of the body; F) fundus transection leaving the fundoplication intact.

The first step of the surgery is to open the phrenoesophageal membrane in order to obtain a complete visualization of the diaphragmatic crus by an anterior and posterior approach. Afterwards, 360° isolation of the abdominal esophagus and esophagogastric junction is performed. The first two short gastric vessels are divided in order to facilitate the Nissen fundoplication. The crus are closed with two or three silk 2/0 stitches. Then a Nissen fundoplication, 5 cm long in a symmetric fashion with three stitches of the same material, is performed over a 34 F bougie. The fundoplication is fixed with a posterior gastropexy to the closed crus with one to two silk 2/0 stitches (Figures 2A and 2B).

In order to perform the sleeve gastrectomy, dissection of the greater curvature is initiated using an advanced coagulation instrument. The dissection begins at the junction between the gastric body and antrum. It continues upwards towards the gastroesophageal junction, ending at the esophagogastric angle. The dissection is completed coming back, and ending towards the pylorus. A 36 F bougie is positioned in the lesser curvature and then we proceed to perform the sleeve gastrectomy starting the transection 2-3 cm proximal to the pylorus, using consecutive 60 mm loads. When gastric transection arrives in front of the lower part of fundoplication, the stapler is positioned laterally for gastric division, leaving a fundus segment of 5 cm for fundoplication (Figures 2C, 2D, 2E and 2F).

The bougie is removed and a methylene blue test is performed.

**FIGURE 3:**
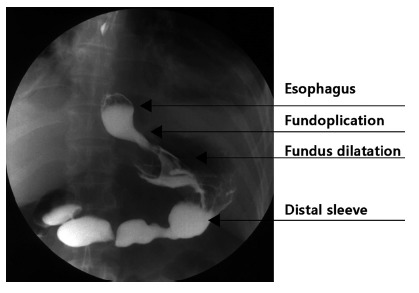
Radiological evaluation with barium sulphate swallow after surgery

Postoperative management starts with early ambulation and oral liquids in the first postoperative day. On the third day, an upper GI series is performed. Figure 3 shows barium swallow evaluation after surgery. With good oral tolerance, we proceed with discharge.

### Statistical analysis

For comparing the results, was used STATA 11 with α of 0.05.

## RESULTS

In group A, 100% (n=15) patients had preoperative manometry demonstrating a hypotensive sphincter, with a mean LES resting pressure of 8.35±1.64 mmHg. All the patients (n=23) showed abnormal 24 h pH monitoring, with a mean exposure to acid time (pH<4), of 13.1±7.09%, and the upper endoscopy showed signs of esophagitis in 80% (n=12) of the patients.

The preoperative study in group B showed a normal manometry and 24 h pH monitoring in 100% of the patients (n=23). The upper endoscopy also showed no signs of esophagitis (Tables 2 and 3).

**TABLE 2:** Esophageal functional studies after laparoscopic sleeve gastrectomy

	Hypotensive LES			Abnormal acid reflux (24 h pHmetry)
	Preop	Postop	Preop	Postop
LSG with Nissen				
(n=15)	15 (100%)	2 (13.3%)	15 (100%)	1(6.66%)
LSG without Nissen				
(n=23)	0	17 (73.9%)	0	15(65.2%)

**TABLE 3:** Gastroesophageal reflux after laparoscopic sleeve gastrectomy

	Reflux symptoms			Erosive esophagitis
	Preop	Postop	Preop	Postop
LSG with Nissen				
(n=15)	15 (100%)	3 (20%)	12 (80%)	3 (20%)
LSG without Nissen				
(n=23)	0	5 (21.7%)	0	4(17.4%)

In group A, the mean operating time was 157±22.13 min and a mean hospital stay of 4.6 days. Postoperative complications were observed in two patients (2/15 patients, 13.3%) corresponding to a seroma and another patient presented with late pneumoperitoneum (8^th^ postoperative day) secondary to vomit and gastric retention. This patient received medical treatment with nasogastric tube for two days, then restarting oral liquid ingestion for another two days. Afterwards he progressed with semi-solid foods with good outcome. There was no mortality in the series. 

 In group B the mean operating time was 87min±15 min. The mean hospital stay was 2.6 days. One patient presented an upper gastrointestinal bleeding which was managed with medical and endoscopic treatment (clips) (1/23, 4.3%). No reoperations and no mortality were observed in this group (Table 1).

In the postoperative follow-up of group A, the mean BMI at six months was 28.44±2.76 kg/m^2^, which corresponds to an EWL% of 61.34%. The mean BMI at 12 months was 26.6±1.7 kg/m^2^ with EWL% of 82.02%. In the 24 h pH monitoring and manometry at three months postoperatively, the LES resting pressures increased in all patients, to a mean of 14.46±1.35 mmHg and times of acid exposure decreased in all patients to a mean of 2.76%±0.35. This represents a normal manometry in 86.6% (n=13) patients, and a normal 24 h pH monitoring in 93.3% (n=14) patients (Tables 2 and 3). 

The postoperative study in group B showed a hypotensive LES in a 73.9% (n=17), abnormal acid reflux in a 65.2% (n=15), reflux symptoms in a 21.7% (n=5) and erosive esophagitis in a 17.4% (n=4, Tables 2 and 3).

**FIGURE 4:**
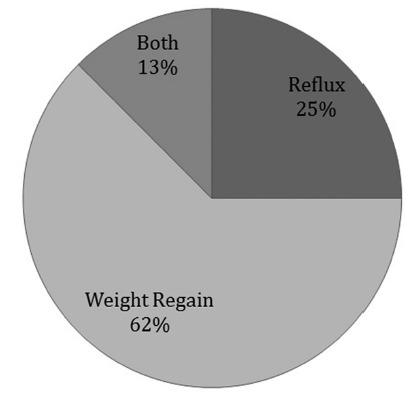
Cause of reoperations

During long-term monitoring and via telephone survey, covering up to nine years after surgery, 40% (n=6) patients required a reoperation. One required reoperation two months after surgery due to intractable epigastric pain nausea, persistent erosive esophago-gastritis with bile reflux, but most (n=3) of these patients were reoperated because of weight regaining. One more patient had regained weight and presented reflux symptoms. An additional patient presented failure of Nissen fundoplication with hiatal hernia recurrence and erosive esophagitis confirmed by endoscopy and barium swallow. This patient continues medical treatment with proton pump inhibitor (Nexium®) up to now, and is under consideration for reoperation but refused surgery. There are two additional patients who refused a new surgery although they have weight regain. In total, 53.3% (n=8) have been re-operated, or have revisional surgery indication for weight regain or symptomatic reflux. The majority of these patients were re-operated before the third year after the initial surgery (range two months to four years). Due to this observation we stopped the indication of this type of surgery (Figure 4).

## DISCUSSION

Studies attempting to elucidate the relationship between gastrectomy and reflux have been varied and present disparate results.

There are physiopathological bases that support both views. On one hand, obesity, increased intragastric pressure and the consequent increased esophagus-gastric pressure gradient, support why reflux could be increased in these patients. This can be associated with the appearance of a hiatal hernia, also more common in obese patients. In this context, sleeve gastrectomy and weight loss has a positive effect, reducing the occurrence of reflux. Other effects that could reduce the presence of reflux are lower acid production and accelerated gastric emptying.

On the other hand, the lower gastric compliance, increased intragastric pressure, decreased LES resting pressure, non-resolution of a hiatal hernia and stenosis in the gastric angle are all reasons underlying higher reflux after surgery^1^ ^,^ ^4^ ^,^ ^5^ ^,^ ^26^ ^,^ ^30^ ^,^ ^41^. A study by Braghetto et al.^6^ showed significant decrease in resting LES pressure in 85% of patients studied in a post-SG series. There are several other publications that support this results^7^ ^,^ ^15^ ^,^ ^24^.

Systematic reviews have not been capable to elucidate this question, mainly by the quality of evidence. However, most recent papers have concluded that LSG is associated with worsening or “de novo” GERD after surgery.

In a systematic review, 13 studies with negative effects in reflux after LSG were found. They included 5953 patients, with mean BMI of 42±4 kg/m^2^ and a mean follow-up of 29±22 months. Only one of these studies was a prospective randomized trial. Reflux presence was evaluated with clinical symptoms in most studies. The results showed a high percentage of persistent reflux after surgery, which came to 84% in a retrospective study Dupree et al.^12^. Thereauxet al.^42^ in their paper conclude that LSG was associated with de novo GERD in over two-thirds of patients, but did not seem to exacerbate existing GERD.

In contrast, other studies showed favorable results after LSG. They included 1863 patients, with mean BMI of 51±13 kg/m^2^ and a mean follow-up of 20±15 months. All studies used clinical assessment scales for reflux^7^ ^,^ ^10^ ^,^ ^15^ ^,^ ^24^. One of these studies was by Daes et al.^10^ who in a prospective study with 382 patients, showed that 94% were symptom free, giving emphasis to the surgical technique, avoiding narrowing in the middle of the stomach and firing the stapler on the front and back of an aligned stomach to prevent rotation or spiraling^10^.

Also, Pallati et al.^28^ in a prospective study of 585 patients, showed improvement in 41%; Santonicola et al.^38^ published a decreased in reflux of 39.2% to 22.5% after the LSG; and Rebecchi et al.^32^ published similar findings^1^.

However, there are two recent reviews by Altieri et al.^1^ and Nadaletto et al.^26^ in which the majority of authors conclude that LSG is associated with worsening of GERD or “de novo” GERD after surgery and improvement is mentioned only by few papers. Other studies report about the appearance of complications of de novo GERD after LSG. Braghetto et al.^4^ studied 231 patients undergoing LSG without symptoms of reflux and normal pre-operative endoscopies, and 23% developed symptoms of reflux, with a 5.5% of endoscopic esophagitis and 1.2% of Barrett’s esophagus^2,3,4,31,^.

 According to the current knowledge laparoscopic Roux-en-Y gastric bypass appears as the best option for treat obese patients and GERD^2^ ^,^ ^31^. However, it is associated with late complications not only due to malnutrition or vitamin deficiencies, but also with marginal ulcers, bleeding, gastro-gastric fistulas and other surgical complications that must be taken into account and compared with long term complications after LSG^8^ ^,^ ^13^ ^,^ ^14^ ^,^ ^17^ ^,^ ^29^ ^,^ ^43^. In this context, in order to avoid the mentioned complications, there are previous studies suggesting combinations of antireflux surgery with sleeve gastrectomy. In addition, some authors have proposed some technical modifications in order to improve postoperative results^9^ ^,^ ^11^ ^,^ ^20^ ^,^ ^21^ ^,^ ^22^ ^,^ ^34^ ^,^ ^35^ ^,^ ^36^ ^,^ ^40^. Haswalli et al.^16^ recommended hiatoplasty and anterior fundoplication to treat gastroesophageal reflux using a dilated fundus after LSG. Le Page et al.^20^ presented the effects of a sleeve gastrectomy associated with fundoplication in patients with symptoms of reflux and delayed gastric emptying. The study included four patients who also had hiatal hernia. A fundoplication in 120^o^ associated with the sleeve gastrectomy was performed. All patients improved in scores of symptoms in a GERD questionnaire. The mean weight was reduced by 11% and the BMI by three points, to a mean of 24.

Meanwhile, Lee et al.^21^ published a series of obese patients undergoing Nissen fundoplication associated with gastric plication. The series consisted of 25 obese patients, mean BMI of 37 and reflux symptoms. The operative time was a mean 146 min. All surgeries were performed laparoscopically. The mean hospital stay was 1.5 days and two patients required re-exploration for complications, representing 8%. During follow-up, the mean BMI was 30.8 at one year, corresponding to a 46.7% EWL. One patient was re-operated at 10 months postoperative due to lack of weight loss. Esophagitis fell from 80% to 20%, and there was an improvement in the symptoms of GERD.

Desart et al.^11^ presented the first reported pilot case series, illustrating that the LINX^®^ device as a safe and effective option in patients with de novo refractory gastroesophageal reflux disease after a laparoscopic sleeve gastrectomy despite appropriate weight loss. Robotic LSG with hiatoplasty and anterior fundoplication has also been performed. 

The reason our group abandoned LSG with concomitant fundoplication was because regain weight was observed early in a great proportion of patients in agreement with other reports. This study used a similar technique as that presented by Le Page et al.^20^ including a limited number of patients, because we routinely indicate laparoscopic Roux-en-Y gastric bypass in obese patients with reflux. This procedure was abandoned mainly due to the unsatisfactory results observed specially in terms of weight regain, which is higher than after Nissen Fundoplication for reflux (8-15%)^39^.

 Himpens et al.^40^ and others have reported the need for revisional surgery after regular sleeve, due to GERD^18^ ^,^ ^19^ ^,^ ^27^ ^,^ ^39^. All these procedures need long-term follow-up in order to have the definitive opinion of their advantages. We think this is not a problem secondary to learning curve because the surgeons who performed the surgeries (IB, LG) have a long experience with antireflux surgery and LSG. There were only two complications (13.3%) after the procedure, which is higher than the complications observed in patients with LSG without antireflux surgery or laparoscopic Roux-en-Y gastric bypass. The explanation for higher complication rate and longer operative time of the procedure is because this procedure involves a combination of two surgeries (fundoplication with crus closure and sleeve gastrectomy) and therefore becomes a riskier operation. In our opinion, this procedure itself has technical limitations in order to obtain optimal results in terms of BMI control. The problem with doing a fundoplication with sleeve is that the fundus is not resected totally and a sizable fundus is required, making these patients at risk for weight regain due to dilatation of this fundus as is shown in Figure 4.

The results during the first year were promising, showing a good weight loss and improvement in the objective studies of reflux. At the long term, the results were discouraging, with more than half of the patients requiring or in need of a new surgery to address the weight regain or the symptoms related to reflux. Even though it appears to be a safe procedure, with no mortality and minimal morbidity, the long-term results show that while trying to address both pathologies with a mixed surgery, we suffered from the downfalls of each individual procedure. 

The limitations of this study are great, and while other similar studies show good results in the short term, we need further studies that address the long-term results to consider this surgery as an option in these patients. 

## CONCLUSIONS

Our study shows good results in the short term; however, it has a high recurrence of both, weight gain and reflux symptoms, in the long-term follow-up. Studies using similar techniques show similar short-term results, but also suffer from various problems. In this sense, we cannot recommend this procedure as primary technique in obese patients with reflux. Currently we perform gastric bypass in obese patients with GERD, and we require more and stronger evidence to clarify what is the best procedure for these patients.

## References

[1] Altieri MS, Pryor AD (2015). Gastroesophageal reflux disease after bariatric procedures Surg Clin North. Am.

[2] Braghetto I, Korn O, Csendes A (2012). Laparoscopic treatment of obese patients with gastroesophageal reflux disease and Barrett's esophagus a prospective study. Obes Surg.

[3] Braghetto I, Csendes A (2016). Prevalence of Barrett's Esophagus in Bariatric Patients Undergoing Sleeve Gastrectomy. Obesity Surgery.

[4] Braghetto I., Csendes A., Korn O., Valladares H., Gonzalez P., Henríquez A (2010). Gastroesophageal Reflux Disease After Sleeve Gastrectomy. Surgical Laparoscopy, Endoscopy & Percutaneous Techniques.

[5] Braghetto I., Csendes A., Lanzarini E., Papapietro K., Cárcamo C., Molina J (2012). Is Laparoscopic Sleeve Gastrectomy an Acceptable Primary Bariatric Procedure in Obese Patients Early and 5-Year Postoperative Results. Surgical Laparoscopy, Endoscopy & Percutaneous Techniques.

[6] Braghetto I., Lanzarini E., Korn O., Valladares H., Molina J., Henriquez A (2009). Manometric Changes of the Lower Esophageal Sphincter After Sleeve Gastrectomy in Obese Patients. Obesity Surgery.

[7] Burgerhart JS1, Schotborgh CA, Schoon EJ, Smulders JF, van de Meeberg PC, Siersema PD, Smout AJ (2014). Effect of sleeve gastrectomy on gastroesophageal reflux. Obes Surg.

[8] Chang PC, Huang CK, Rajan M, Hsin MC (2016). Revision with Totally Hand-Sewn Gastrojejunostomy and Vagotomy for Refractory Marginal Ulcer after Laparoscopic Roux-en-Y Gastric Bypass. Obes Surg.

[9] da Silva LE, Alves MM, El-Ajouz TK, Ribeiro PC, Cruz RJ (2015). Laparoscopic Sleeve-Collis-Nissen Gastroplasty a Safe Alternative for Morbidly Obese Patients with Gastroesophageal Reflux Disease.Obes. Surg.

[10] Daes J, Jimenez ME, Said N, Dennis R (2014). Improvement of gastroesophageal reflux symptoms after standardized laparoscopic sleeve gastrectomy. Obes Surg.

[11] Desart K, Rossidis G, Michel M, Lux T, Ben-David K (2015). Gastroesophageal Reflux Management with the LINX(r) System for Gastroesophageal Reflux Disease Following Laparoscopic Sleeve Gastrectomy. J Gastrointest Surg.

[12] DuPree CE, Blair K, Steele SR, Martin MJ (2014). Laparoscopic sleeve gastrectomy in patients with preexisting gastroesophageal reflux disease a national analysis.JAMA. Surg.

[13] EL-Hayek K, Timratana P, Shimizu H (2012). marginal ulcer after Roux-en-Y Gastric bypass: what we really learned. Sur Endosc.

[14] Gasteyger C, Suter M, Gaillard RC (2008). Nutritional deficiencies after Roux-en-Y gastric bypass for morbid obesity often cannot be prevented by standard multivitamin supplementation. Am J Clin Nutr.

[15] Gorodner V, Buxhoeveden R, Clemente G, Solé L, Caro L, Grigaites A (2015). Does laparoscopic sleeve gastrectomy have any influence on gastroesophageal reflux disease Preliminary results. Surg Endosc.

[16] Hawasli A, Reyes M, Hare B, Meguid A, Harriott A, Almahmeed T, Thatimatla N, Sapunar S (2016). Can morbidly obese patients with reflux be offered laparoscopic sleeve gastrectomy A case report of 40 patients. Am J Surg.

[17] Higa K, Ho T, Tercero F, Yunus T, Boone KB (2011). Laparoscopic Roux-en-Y gastric bypass: 10-year follow-up. Surg Obes Relat Dis.

[18] Himpens J, Dapri G, Cadière GB (2006). A prospective randomized study between laparoscopic gastric banding and laparoscopic isolated sleevegastrectomy: results after 1 and 3 years. Obes Surg.

[19] Howard DD, Caban AM, Cendan JC, Ben-David K (2011). Gastroesophageal reflux after sleeve gastrectomy in morbidly obese patients. Surg Obes Relat Dis.

[20] Le Page P, Martin D (2015). Laparoscopic Partial Sleeve Gastrectomy with Fundoplication for Gastroesophageal Reflux and Delayed Gastric Emptying. World J Surg.

[21] Lee W., Han M., Ser K., Tsou J., Chen J., Lin C (2014). Laparoscopic Nissen Fundoplication with Gastric Plication as a Potential Treatment of Morbidly Obese Patients with GERD, First Experience and Results. Obesity Surgery.

[22] Mahawar KK, Carr WR, Jennings N, Balupuri S, Small PK (2015). Simultaneous sleeve gastrectomy and hiatus hernia repair: a systematic review. Obes Surg.

[23] Melissas J., Braghetto I., Molina J., Silecchia G., Iossa A., Iannelli A., Foletto M (2015). Gastroesophageal Reflux Disease and Sleeve Gastrectomy. Obesity Surgery.

[24] Mion F, Tolone S, Garros A, Savarino E, Pelascini E, Robert M, Poncet G, Valette PJ, Marjoux S, Docimo L, Roman S (2016). High-resolution Impedance Manometry after Sleeve Gastrectomy: Increased Intragastric Pressure and Reflux are Frequent Events. Obes Surg.

[25] Mion F, Dargent J (2014). Gastro-oesophageal reflux disease and obesity: Pathogenesis and response to treatment. Best Practice & Research Clinical Gastroenterology.

[26] Nadaleto B., Herbella F., Patti M (2016). Gastroesophageal reflux disease in the obese Pathophysiology and treatment. Surgery.

[27] Nelson L, Teixeira AF, Jawad MA (2015). Robotic sleeve gastrectomy, hiatal hernia repair and anterior fundoplication in a patient with symptomatic GERD. Surg Obes Relat Dis.

[28] Pallati PK, Shaligram A, Shostrom VK, Oleynikov D, McBride CL, Goede MR (2014). Improvement in gastroesophageal reflux disease symptoms after various bariatric procedures review of the Bariatric Outcomes Longitudinal Database. Surg Obes Relat Dis.

[29] Paroz A, Calmes JM, Giusti V (2006). Internal hernia after laparoscopic Roux-en-Y Gastric bypass for morbid obesity a continuous challenge in bariatric surgery. Obes Surg.

[30] Prachand V (2010). Gastroesophageal reflux disease and severe obesity: Fundoplication or bariatric surgery. World Journal of Gastroenterology.

[31] Praveendra P, Gomez RM, Kumar S, senthilnathan P, Parthasarathi R, Rjapandian S, Palanivelu C (2016). Laparoscopic Undo of fundoplication with Roux-en-Y gastric bypass in a morbidly obese patient with prior Nissen´s fundoplication: a video report. Obes Sur.

[32] Rebecchi F1, Allaix ME, Giaccone C, Ugliono E, Scozzari G, Morino M (2014). Gastroesophageal reflux disease and laparoscopic sleeve gastrectomy a physiopathologic evaluation.Ann. Surg.

[33] RIBEIRO Jeany Borges, Silva (2015). LOWER ESOPHAGEAL SPHINCTER PRESSURE MEASUREMENT UNDER STANDARDIZED INSPIRATORY MANEUVEURS. ABCD, arq. bras. cir. dig.

[34] Ruscio S, Abdelgawad M, Badiali D, Iorio O, Rizzello M, Cavallaro G, Severi C, Silecchia G (2015). Simple versus reinforced cruroplasty in patients submitted to concomitant laparoscopic sleevegastrectomy: prospective evaluation in a bariatric center of excellence. Surg Endosc.

[35] Samakar K, McKenzie TJ, Tavakkoli A, Vernon AH, Robinson MK, Shikora SA (2016). The Effect of Laparoscopic Sleeve Gastrectomy with Concomitant Hiatal Hernia Repair on Gastroesophageal Reflux Disease in the Morbidly Obese. Obes Surg.

[36] Sánchez-Pernaute A, Talavera P, Pérez-Aguirre E, Domínguez-Serrano I, Rubio MÁ, Torres A (2016). Technique of Hill's Gastropexy Combined with Sleeve Gastrectomy for Patients with Morbid Obesity andGastroesophageal Reflux Disease or Hiatal. HerniaObesSurg.

[37] SANTO Marco Aurelio (2015). ENDOSCOPIC CHANGES RELATED TO GASTROESOPHAGEAL REFLUX DISEASE: COMPARATIVE STUDY AMONG BARIATRIC SURGERY PATIENTS. ABCD, arq. bras. cir. dig.

[38] Santonicola A., Angrisani L., Cutolo P., Formisano G., Iovino P (2014). The effect of laparoscopic sleeve gastrectomy with or without hiatal hernia repair on gastroesophageal reflux disease in obese patients. Surgery for Obesity and Related Diseases.

[39] Siewert JR, Isolauri J, Feussner H (1989). Reoperation following failed fundoplication. World J Surg.

[40] Soricelli E1, Casella G, Rizzello M, Calì B, Alessandri G (2010). Basso NInitial experience with laparoscopic crural closure in the management of hiatal hernia in obese patients undergoing sleeve gastrectomy. Obes Surg.

[41] Stenard F (2015). Laparoscopic sleeve gastrectomy and gastroesophageal reflux. World Journal of Gastroenterology.

[42] Thereaux J, Barsamian C, Bretault M, Dusaussoy H, Lamarque D, Bouillot JL, Czernichow S, Carette C (2016). pH monitoring of gastro-oesophageal reflux before and after laparoscopic sleeve gastrectomy. Br J Surg.

[43] Zak Y, Petrusa E, Gee DW (2015). Laparoscopic Roux-en-Y gastric bypass patients have an increased lifetime risk of repeat operations when compared to laparoscopic sleeve gastrectomy patients. Surg Endosc.

